# Design and Simulation of a High-Performance GaN Vertical Merged P-i-N/Schottky (MPS) Diode with Multi-Drift-Layer and Field-Plate Termination

**DOI:** 10.3390/mi17060722

**Published:** 2026-06-14

**Authors:** Yun Seop Yu, Saebin Yoon, Jong Hyeok Oh

**Affiliations:** ICT & Robotics Engineering, Semiconductor Convergence Engineering, AISPC Laboratory and IITC, Hankyong National University, 327 Jungang-ro, Anseong-si 17579, Gyenggi-do, Republic of Korea; sabinmiin@naver.com (S.Y.); rnjsdlr7@hknu.ac.kr (J.H.O.)

**Keywords:** GaN power diode, merged PiN Schottky (MPS), TCAD, mixed-mode TCAD, power semiconductor

## Abstract

This paper presents the design, structural optimization, and two-dimensional (2D) technology computer-aided design (TCAD) simulation of a gallium nitride (GaN) vertical Merged P-i-N/Schottky (MPS) diode incorporating a multi-drift-layer doping profile, composite SiO_2_/Si_3_N_4_ passivation, and field-plate (FP) termination. The proposed device is constructed on an n^+^-GaN substrate with a three-sub-layer n-type drift region and a p-GaN/p^+^-GaN anode region. Systematic TCAD simulations are performed to investigate the dependences of key performance metrics—including knee voltage (*V*_knee_), specific on-resistance (*R*_on_), breakdown voltage (*BV*), reverse leakage current (*J*_leak_), and Baliga’s figure of merit (*BFOM*)—on the Schottky metal work function, multi-drift-layer doping concentration, drift-layer thickness, Schottky-to-PN contact length ratio (*γ*_w_), operating temperature, and reverse recovery switching transients. Results demonstrate that the MPS architecture effectively decouples forward conduction loss from reverse blocking capability, overcoming the conventional *R*_on_–*BV* trade-off. The optimal doping profile (*n*_mm_ = 2 × 10^15^, *n*_m_ = 2 × 10^15^, *n* = 1 × 10^16^ cm^−3^) achieves a *BFOM* of ~31.97 GW·cm^−2^ with *BV* ≈ 5.98 kV and *R*_on_ ≈ 1.12 mΩ·cm^2^. Joint doping–thickness optimization further identifies a graded doping profile (*n*_mm_ = 2 × 10^15^, *n*_m_ = 5 × 10^15^, *n* = 1 × 10^16^ cm^−3^) combined with layer thicknesses (*T*_nmm_, *T*_nm_, *T*_n_) = (4.49, 5, 20) μm as the overall optimum, achieving *BFOM* = 55.36 GW·cm^−2^ (*BV* = 6.61 kV, *R*_on_ = 0.79 mΩ·cm^2^)—a +73% improvement, governed by the punch-through/field-stop design principle. The optimal contact ratio of γ_w_ = 1.33 yields a *BFOM* of 38.71 GW·cm^−2^. Temperature analysis confirms a positive *BV* temperature coefficient due to drift-region-limited avalanche breakdown, and the *BFOM* improves monotonically from 33.31 to 37.82 GW·cm^−2^ between 200 K and 450 K. Mixed-mode switching simulations show that increasing *γ*_w_ substantially reduces reverse recovery charge (*Q*_rr_), demonstrating the strong potential of the proposed MPS diode for high-voltage, high-frequency, and high-temperature power electronic applications.

## 1. Introduction

Wide-bandgap (WBG) semiconductor materials have attracted intense research interest for next-generation power electronic applications due to their superior physical properties compared with conventional silicon (Si) [1,2]. Among WBG semiconductors, gallium nitride (GaN) stands out owing to its wide bandgap (~3.4 eV), high critical electric field (~3.3 MV/cm), high electron mobility, and high thermal conductivity [3,4]. These properties place GaN well above Si and on par with or superior to silicon carbide (SiC) in terms of Baliga’s figure of merit (*BFOM*), making it an attractive material platform for power rectifiers and switches operating at high voltage, high current, and elevated temperatures [5,6].

Vertical GaN power diodes are particularly attractive for high-power applications because the vertical current path allows the drift region to independently sustain the blocking voltage, enabling higher breakdown voltage (*BV*) and current density than lateral device architectures [7,8]. Among vertical GaN rectifiers, Schottky barrier diodes (SBDs) offer low knee voltage (*V*_knee_) and fast unipolar switching, but suffer from high reverse leakage current (*J*_leak_) and limited *BV* due to electric-field crowding at the metal–semiconductor interface [9,10]. In contrast, GaN P-i-N (PiN) diodes achieve excellent reverse blocking capability with near-ideal avalanche behavior, but exhibit a significantly higher *V*_knee_ and large on-resistance (*R*_on_) at low current densities due to the bipolar turn-on voltage [11,12].

The Merged P-i-N/Schottky (MPS) diode architecture—also referred to as a Junction Barrier Schottky (JBS) diode in some literature—was originally developed for SiC and has more recently been investigated for GaN [13,14]. The MPS structure integrates Schottky and ohmic contacts at the anode, such that forward current flows primarily through the low-resistance Schottky path, while embedded p-GaN regions shield the Schottky interface from high electric fields under reverse bias [15,16]. This dual-path configuration enables simultaneous achievement of low *V*_knee_, reduced *R*_on_, and high *BV*, effectively relaxing the fundamental *R*_on_–*BV* trade-off inherent to conventional unipolar and bipolar devices [17,18].

Field-plate (FP) termination has been widely adopted in GaN power devices to redistribute the electric field at device edges, suppressing premature edge breakdown and improving *BV* [19,20]. Composite dielectric passivation layers, such as SiO_2_/Si_3_N_4_ stacks, further reduce surface leakage and trap-related degradation, contributing to improved long-term device reliability [21,22]. In addition, engineering the drift-layer doping profile through a multi-layer approach—rather than a uniform single layer—allows simultaneous optimization of forward conduction and reverse blocking by tailoring the electric field distribution within the drift region [23].

Despite the growing body of experimental and simulation literature on GaN SBDs, PiN diodes, and MPS structures, a comprehensive and systematic design optimization of a vertical GaN MPS diode combining all three elements—multi-drift-layer profile, composite passivation, and FP termination—remains limited. Specifically, the independent and combined influences of the Schottky barrier height (via metal work function), multi-drift-layer doping profile, Schottky-to-PN contact length ratio (*γ*_w_), temperature, and switching dynamics on the comprehensive set of performance metrics have not been systematically quantified within a unified device framework [24,25].

In this work, we present a detailed 2D TCAD design and simulation study of a GaN vertical MPS diode incorporating a three-sub-layer n-type drift region, composite SiO_2_/Si_3_N_4_ passivation, and anode field-plate termination using the TCAD simulator [26,27]. We systematically investigate the effects of: (1) Schottky metal work function; (2) multi-drift-layer doping concentration; (3) Schottky-to-PN contact length ratio; (4) operating temperature; and (5) reverse recovery switching transients; and (6) drift-layer thickness, including the joint doping-thickness optimum. The results provide a clear design guideline for realizing high-voltage, low-loss GaN MPS diodes suitable for next-generation power electronics.

## 2. Device Structure and Simulation Methodology

### 2.1. Proposed MPS Diode Structure

**Figure 1:**
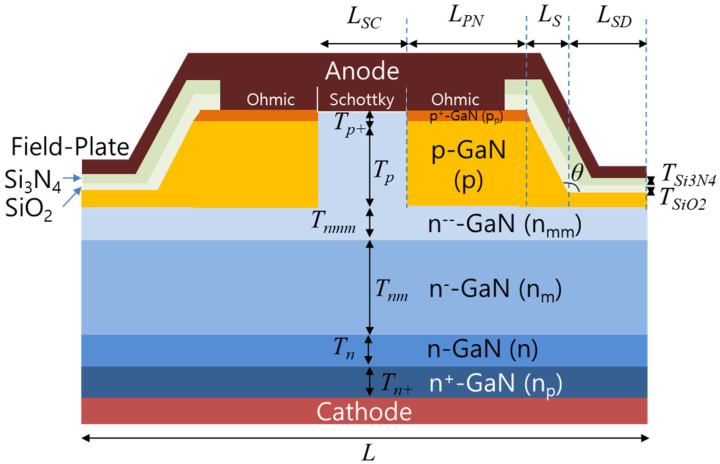
Cross-sectional schematic of the proposed gallium nitride (GaN) vertical merged P-i-N/Schottky (MPS) diode.

Figure 1 illustrates the cross-sectional schematic of the proposed GaN vertical MPS diode. The device is built on an n^+^-GaN substrate serving as the cathode, upon which a multi-layered n-type drift region is epitaxially grown. The drift region consists of three sub-layers from bottom to top: n-GaN (*T*_n_ = 5 μm), n^−^-GaN (*T*_nm_ = 20 μm), and n^− −^-GaN (*T*_nmm_ = 4.49 μm). Above the drift region, a p-GaN layer (*T*_p_ = 0.5 μm, variable) is formed, capped with a thin p^+^-GaN contact layer (*T*_p+_ = 0.01 μm) at the mesa-etched anode regions.

The anode electrode contacts both ohmic (p^+^-GaN) and Schottky regions, realizing the MPS configuration. The Schottky contact of length *L*_SC_ = 1.5 μm is located at the center of the anode cell, flanked by p-GaN regions of length *L*_PN_ = 2.1 μm on each side. A spacing *L*_S_ = 0.15 μm separates the Schottky contact from the p-GaN mesa sidewall. The composite passivation layer—consisting of SiO_2_ (*T*_SiO2_ = 0.1 μm) deposited first, followed by Si_3_N_4_ (*T*_Si3N4_ = 0.1 μm)—covers the mesa sidewalls and the field-plate dielectric region. The field plate extends horizontally over the passivation layer for a length *L*_SD_ = 1.25 μm beyond the anode metal edge.

The complete structural parameters are summarized in Table 1. The combination of the MPS architecture, multi-drift-layer doping, composite passivation, and field-plate termination is designed to simultaneously achieve for reduced *V*_knee_, minimized *R*_on_, elevated *BV* through electric field redistribution, and suppressed leakage current.

### 2.2. Simulation Methodology

All simulations were performed using the 2D TCAD Synopsys Sentaurus-Device simulator [26]. The key physical models employed include: (1) Fermi-Dirac carrier statistics; (2) concentration-dependent Shockley–Read–Hall (SRH) recombination [28]; (3) band-to-band tunneling (BTBT) for reverse-bias leakage at the Schottky junction via the non-local tunneling model; (4) Selberherr impact ionization model with non-local electric field treatment for avalanche breakdown analysis [29]; and (5) carrier mobility described by the Masetti doping-dependent model [30] combined with Caughey–Thomas-type high-field velocity saturation [27]. In addition, incomplete ionization of the dopants and a bulk deep-level trap density of 1.25 × 10^15^ cm^−3^ (*E*_C_ − 0.7 eV), representative of native point defects in epitaxial GaN, are included in all simulations, so that the influence of point defects on the electrostatics, leakage, and breakdown is explicitly captured. It should be noted that, in actual GaN epitaxial growth, the mobility at low doping levels (<1 × 10^17^ cm^−3^) is influenced not only by impurity scattering but, more importantly, by compensating point defects such as carbon-related deep acceptors and gallium-vacancy complexes [31,32,33,34]: increasing the doping concentration at low doping levels compensates the point defects and enhances carrier screening, so that the measured mobility can actually increase with doping, with a maximum typically near 10^16^–10^17^ cm^−3^. Because the doping-dependent mobility model does not account for this compensation effect, the simulated mobilities of the lightly doped layers represent an idealized, uncompensated upper bound; the impact of this idealization is discussed in Section 3.2.

The Schottky barrier height (*ϕ*_B_) is set according to the specified metal *WF*: *ϕ*_B_ = *WF* − *χ*_GaN_, where *χ*_GaN_ = 4.1 eV is the GaN electron affinity [35]. Three *WF* values—4.5, 5.0, and 5.2 eV—are investigated, corresponding to *ϕ*_B_ of approximately 0.4, 0.9, and 1.1 eV, respectively. For the forward current-voltage (*I-V*) simulations, the anode voltage is swept from 0 to 10 V. For reverse *I-V* simulations, the anode voltage is swept negatively until device breakdown. Current density *J* [A/cm^2^] is computed from the 2D TCAD current *I* [A/μm] (per unit *z*-width) normalized to the simulated device length of 5 μm: *J* = *I*/(5 × 10^−8^) [A/cm^2^].

The specific *R*_on_ is evaluated at two bias points: *R*_on1_ (differential resistance *dV*/*dI* at *V* = 2.5 V) and *R*_on2_ (differential resistance *dV*/*dI* at *V* = 8 V), both converted to [mΩ·cm^2^], *V*_knee_ is defined as the anode voltage at which the forward current density reaches 200 A/cm^2^. *J*_leak_ is reported as *J* [A/cm^2^] at a reverse anode voltage of −10 V for device-level comparison. *BV* is uniformly defined throughout this work as the reverse anode voltage at which the reverse current density reaches *J* = 2 A/cm^2^. This identical criterion is applied to all devices (SBDs with different work functions, the PN diode, and all MPS diode configurations), and the corresponding horizontal reference line is shown in all reverse *I-V* figures. *BFOM* is computed as *BFOM* = *BV*^2^/*R*_on_ [5,6].

## 3. Results and Discussion

### 3.1. Work Function Dependence

**Figure 2:**
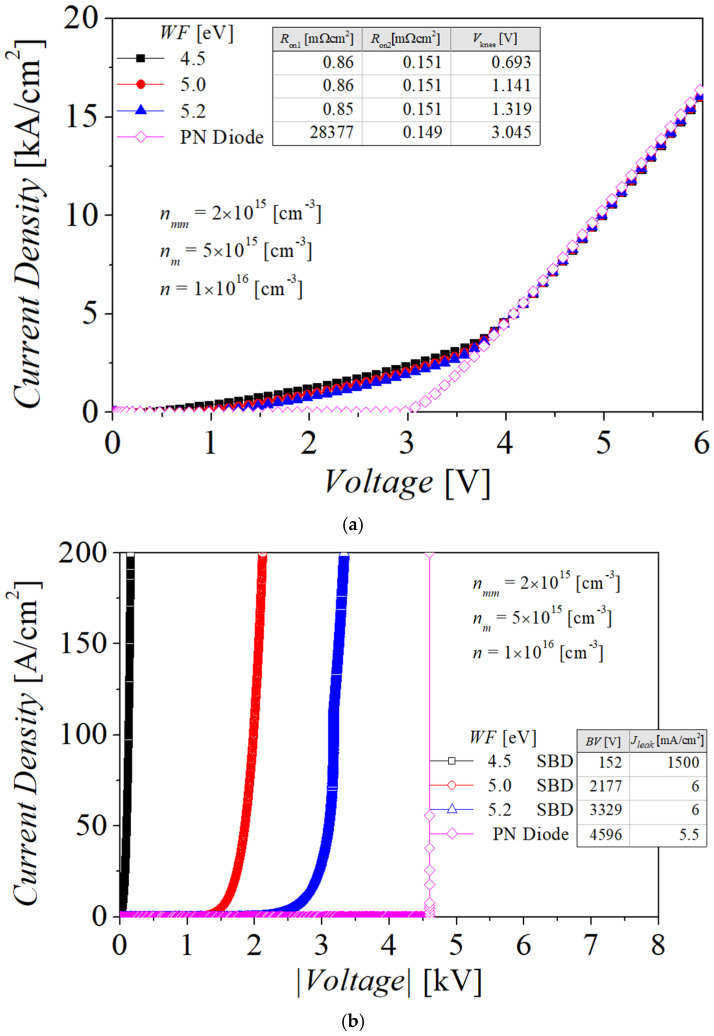
(**a**) Forward and (**b**) reverse *I-V* characteristics of the three Schottky diodes for metal work functions of *WF* = 4.5, 5.0, and 5.2 eV, and a PN diode for comparison for comparison. All devices are simulated with an identical multi-drift-layer doping profile (*n*_mm_ = 2 × 10^15^, *n*_m_ = 5 × 10^15^, and *n* = 1 × 10^16^ cm^−3^).

Figure 2 compares the forward and reverse *I-V* characteristics of three Schottky diodes with different metal work functions (*WF* = 4.5, 5.0, and 5.2 eV) and a conventional PN diode. Table 2 summarizes the forward and reverse performance metrics extracted from Figure 2. All devices were simulated using an identical multi-drift-layer doping profile of *n*_mm_ = 2 × 10^15^, *n*_m_ = 5 × 10^15^, and *n* = 1 × 10^16^ cm^−3^, ensuring that the observed performance differences arise primarily from the contact type and carrier transport mechanism rather than from variations in the drift region. Filled squares, filled circles, filled triangles, and open diamonds denote the *I-V* characteristics of Schottky diodes with *WF*s of 4.5, 5.0, and 5.2 eV, and the PN diode, respectively.

**Table 2:** Forward and reverse performance metrics as a function of metal work function.

*WF* [eV]	Type	*R*_on1_ [mΩ·cm^2^]	*R*_on2_ [mΩ·cm^2^]	*V*_knee_ [V]	*BV* [V]
4.5	SBD	0.86	0.151	0.693	152
5.0	SBD	0.86	0.151	1.141	2177
5.2	SBD	0.85	0.151	1.319	3329
—	PN Diode	28,377	0.149	3.045	4596

As shown in Figure 2a, the forward *I-V* characteristics of the Schottky diodes are strongly dependent on the metal work function. A lower work function reduces *ϕ*_B_, resulting in a smaller *V*_knee_ at low forward bias [35,36]. In contrast, the PN diode exhibits a significantly higher *V*_knee_ and a much larger *R*_on1_, reflecting the inherent voltage drop associated with bipolar junction turn-on. Notably, as indicated in Table 2, *R*_on1_ and *R*_on2_ remain nearly constant for all Schottky diodes, regardless of the work function, demonstrating that the high-current on-state resistance is dominated by the drift-layer resistance rather than by the Schottky contact.

Figure 2b highlights the reverse *I-V* characteristics and breakdown behavior. The PN diode shows excellent reverse blocking capability with low leakage current, which is characteristic of a junction-controlled device. In contrast, conventional Schottky diodes typically suffer from limited *BV* due to strong electric-field crowding at the metal–semiconductor interface [10,19]. However, in the present devices, all Schottky diodes achieve *BV*s in the kilovolt range, comparable to that of the PN diode. This improvement is attributed to the multi-drift-layer structure, which effectively redistributes the electric field into the bulk drift region, thereby shifting the breakdown mechanism from being Schottky-contact-limited to drift-region-limited [23]. Moreover, increasing the metal work function further enhances *BV* and suppresses *J*_leak_ by increasing *ϕ*_B_ [35].

These results clearly illustrate the intrinsic trade-off between *R*_on_ and *BV* in power devices. The PN diode achieves high *BV* at the expense of a large *R*_on_, particularly in the low- to moderate-current regime, whereas a Schottky diode offers low *R*_on_ but is traditionally limited in *BV* [17,18]. The MPS diode structure effectively overcomes this limitation by combining a Schottky conduction path with a drift-layer-supported blocking mechanism. As a result, the MPS diode maintains a low *V*_knee_ and reduced *R*_on_, characteristic of unipolar Schottky conduction, while simultaneously achieving a high *BV* comparable to that of a PN diode. This decoupling of forward conduction loss from reverse blocking capability relaxes the conventional *R*_on_–*BV* trade-off and underscores the necessity of the MPS structure for high-voltage, low-loss power device applications [15,16].

**Figure 3:**
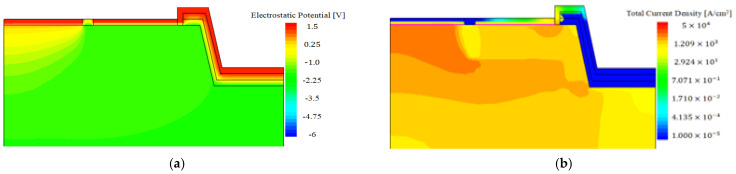
(**a**) Electrostatic potential distribution and (**b**) total current density distribution of the proposed MPS diode with the optimized multi-drift-layer doping profile (*n*_mm_ = 2 × 10^15^, *n*_m_ = 2 × 10^15^, and *n* = 1 × 10^16^ cm^−3^) and a fixed *WF* of 5.2 eV, under a forward anode bias of *V* = +3 V.

**Figure 4:**
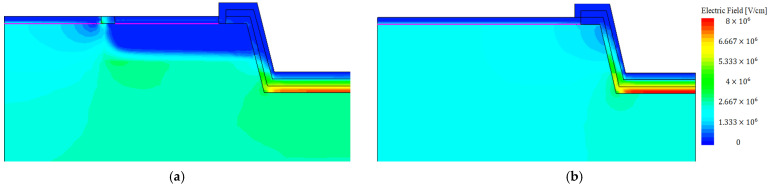
Electric-field distributions of (**a**) the proposed MPS diode and (**b**) the conventional Schottky diode (without the embedded p-GaN regions) for the same multi-drift-layer doping profile (*n*_mm_ = 2 × 10^15^, *n*_m_ = 2 × 10^15^, and *n* = 1 × 10^16^ cm^−3^) and a fixed *WF* of 5.2 eV, under a reverse anode bias of *V* = −5 kV.

To physically substantiate the carrier-transport and field-redistribution mechanisms of the proposed MPS architecture, Figure 3 and Figure 4 present the internal distributions of key physical quantities. Figure 3a,b show the electrostatic potential and the total current density under forward bias (*V* = +3 V). The potential map confirms that the forward bias is correctly localized at the Schottky junction, while the current-density map demonstrates parallel dual-path conduction: high current density flows from the anode through both the central Schottky contact and the lateral p^+^-GaN ohmic contacts, directly visualizing the dual-path conduction mechanism of the MPS architecture. Figure 4a,b compare the reverse-bias (*V* = −5 kV) electric-field distributions of the proposed MPS diode and the conventional Schottky diode (i.e., the same drift structure but without the embedded p-GaN regions), respectively. In the MPS diode [Figure 4a], the embedded p-GaN regions effectively shield the Schottky/GaN interface and redistribute the high-field region into the n^− −^-GaN layer beneath the p-GaN junctions, whereas in the conventional Schottky diode [Figure 4b] the high field is concentrated directly at the Schottky/GaN interface across the entire anode area—the well-known origin of premature Schottky-contact-limited breakdown. This field-redistribution mechanism is the direct physical origin of the elevated *BV* reported in Table 2, Table 3 and Table 4.

### 3.2. Multi-Drift-Layer Doping Concentration Dependence in the Proposed MPS Diode

**Figure 5:**
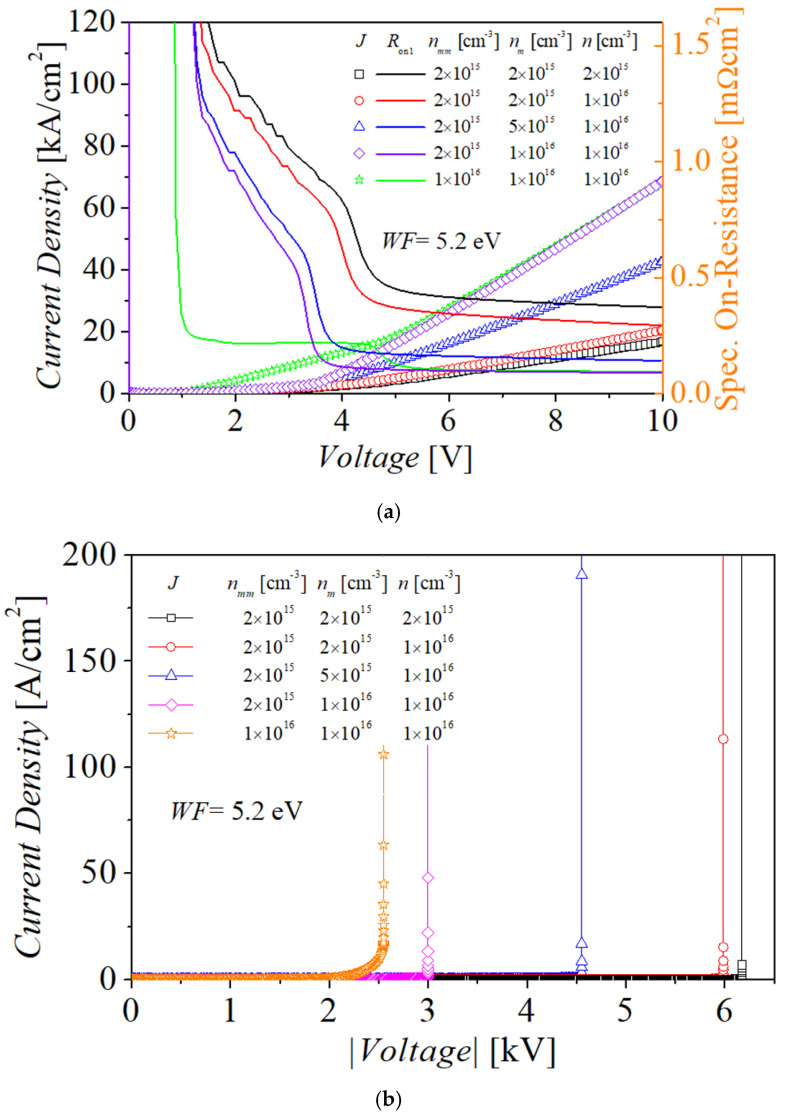
(**a**) Forward and (**b**) reverse *I-V* characteristics of the MPS diodes at a fixed *WF* of 5.2 eV, employing four different multi-drift-layer doping profiles.

Figure 5 compares the forward and reverse *I-V* characteristics of MPS diodes employing four different multi-drift-layer doping profiles, all evaluated at a fixed *WF* of 5.2 eV. From these characteristics, the key performance metrics—including *V*_knee_, *R*_on_, *BV*, *J*_leak_, and *BFOM*—are extracted and summarized in Table 3. Because all devices share the same MPS architecture and Schottky contact, the observed variations can be directly attributed to differences in the drift-layer doping configuration.

As shown in Figure 5a, the forward *I-V* characteristics exhibit systematic changes with the doping profile. Increasing the drift-layer doping concentration reduces both *V*_knee_ and *R*_on_ by lowering the series resistance in the current path [23]. This trend is clearly reflected in Table 3, where *R*_on_ decreases monotonically with higher drift-layer doping. However, excessively high doping levels significantly increase reverse leakage and degrade the blocking capability.

The reverse *I-V* characteristics in Figure 5b reveal the complementary effect of the drift-layer doping on *BV*. Lightly doped profiles achieve higher *BV* due to reduced electric-field peak intensity within the drift region, while more heavily doped structures suffer from premature breakdown [5,17]. This behavior highlights the fundamental *R*_on_–*BV* trade-off governed by drift-layer design.

**Table 3:** *BFOM* performance comparison across multi-drift-layer doping configurations in the MPS diode (*WF* = 5.2 eV).

*n*_mm_ [cm^−3^]	*n*_m_ [cm^−3^]	*n* [cm^−3^]	*V*_knee_ [V]	*R*_on1_ [mΩ·cm^2^]	*BV* [V]	*J*_leak_ [mA/cm^2^]	*BFOM* [GW/cm^2^]
1 × 10^14^	1 × 10^14^	1 × 10^14^	4.411	23.30	6673	0.16	1.91
1 × 10^15^	1 × 10^15^	1 × 10^15^	1.875	2.415	6551	0.41	17.77
2 × 10^15^	2 × 10^15^	2 × 10^15^	1.365	1.20	6170	0.55	31.72
2 × 10^15^	2 × 10^15^	1 × 10^16^	1.352	1.12	5984	0.55	31.97
2 × 10^15^	5 × 10^15^	1 × 10^16^	1.319	0.86	4560	0.55	24.18
2 × 10^15^	1 × 10^16^	1 × 10^16^	1.307	0.77	2994	0.55	11.64
1 × 10^16^	1 × 10^16^	1 × 10^16^	1.006	0.22	2545	1.10	29.44

Among the four profiles examined, the doping configuration with *n*_mm_ = 2 × 10^15^, *n*_m_ = 2 × 10^15^, and *n* = 1 × 10^16^ cm^−3^, exhibits the most balanced performance. This structure achieves a moderate *V*_knee_ of approximately 1.35 V, a low *R*_on_ of about 1.12 mΩ·cm^2^, and a high *BV* of approximately 6 kV. As a result, it delivers the highest *BFOM*, approximately 31.97 GW·cm^−2^, among all investigated cases.

In contrast, more lightly doped configurations improve *BV* but suffer from a substantial increase in *R*_on_, whereas more heavily doped profiles reduce *R*_on_ at the expense of severely degraded *BV*. These results confirm that the optimal performance of an MPS diode cannot be achieved through uniform or extreme drift-layer doping, but instead requires a carefully engineered multi-drift-layer profile [23].

Overall, this optimization study demonstrates that the MPS diode effectively relaxes the conventional *R*_on_–*BV* trade-off by decoupling forward conduction and reverse blocking mechanisms. The Schottky contact ensures low turn-on voltage and reduced conduction loss, while the optimized multi-drift-layer structure sustains high breakdown voltage through electric-field redistribution. Consequently, the identified optimal doping profile provides a clear design guideline for realizing high-voltage, low-loss MPS diodes suitable for next-generation power electronic applications. Among the investigated doping profiles, the structure with *n*_mm_ = 2 × 10^15^, *n*_m_ = 2 × 10^15^, and *n* = 1 × 10^16^ cm^−3^ achieves the highest *BFOM* and represents the best trade-off between *R*_on_ and *BV*. Accordingly, all subsequent analyses in Section 3.3 and beyond were carried out using this optimized doping profile with a fixed *WF* = 5.2 eV to ensure a consistent evaluation of device performance.

It is noteworthy that the uniformly high-doped profile (*n*_mm_ = *n*_m_ = *n* = 1 × 10^16^ cm^−3^) achieves a *BFOM* of 29.44 GW/cm^2^, which is only 8% below the optimal graded profile (31.97 GW/cm^2^) despite a 2.4× lower *BV*. This apparent paradox is a consequence of the quadratic-vs-inverse-quadratic dependence of *BFOM* on *BV* and *R*_on_: as the drift doping is increased uniformly from 2 × 10^15^ to 1 × 10^16^ cm^−3^, *R*_on1_ decreases by a factor of ~5.5× (from 1.20 to 0.22 mΩ·cm^2^), which almost exactly compensates for the ~5.9× reduction in *BV*^2^ (from 6170^2^ to 2545^2^). The net *BFOM* is therefore reduced by less than 10%. However, the absolute *BV* of 2545 V is below the practical requirement for the medium-voltage (≥3.3 kV class) power-electronic applications targeted by this work, so the uniformly high-doped configuration is not preferred despite its competitive *BFOM*. This observation highlights a general limitation of using *BFOM* alone as a design metric: an additional constraint on the absolute BV is required for high-voltage device design.

To further explore the low-doping regime, we extended the uniform-doping sweep in Table 3 to include 1 × 10^14^ and 1 × 10^15^ cm^−3^. The results show that *BV* is essentially saturated across the entire low-doping range—*BV* = 6673 → 6551 → 6170 V for doping = 1 × 10^14^ → 1 × 10^15^ → 2 × 10^15^ cm^−3^ (only ~8% variation across a 20× change in doping). This saturation indicates that the device is already fully punched-through at 2 × 10^15^ cm^−3^, with the depletion region filling the entire drift stack. Meanwhile, *R*_on1_ degrades by ~19× (from 1.20 to 23.30 mΩ·cm^2^) and *V*_knee_ rises from 1.365 to 4.411 V due to series-resistance domination of the current-density definition point. As a result, *BFOM* collapses from 31.72 to 1.91 GW/cm^2^—a 16× degradation. We conclude that 2 × 10^15^ cm^−3^ represents a practical lower bound for the lightly-doped layer in the present drift geometry; operation below this value is not beneficial unless the drift stack is also substantially thickened to raise the punch-through-limited *BV* ceiling.

In actual epitaxial material, compensation by point defects further reduces both the carrier concentration and the mobility in the 10^14^–10^15^ cm^−3^ range [31,32,33,34], so the *R*_on_ values in Table 3 for these cases are best-case estimates and the practical lower bound of 2 × 10^15^ cm^−3^ is, if anything, conservative. The overall conclusions are robust to this idealization, since *BV* is insensitive to mobility and the optimum design conducts mainly through the heavily doped n layer (1 × 10^16^ cm^−3^), where compensation is weak and the mobility model is most reliable.

Importantly, the doping ranking established in this section is conditional on the default drift-layer thicknesses. The complete picture—including the joint doping–thickness optimum, which uncovers a configuration superior to the doping-only optimum reported above—is established in the new Section 3.6.

### 3.3. Schottky-to-PN Contact Length Ratio Dependence of the Proposed MPS Diode

**Figure 6:**
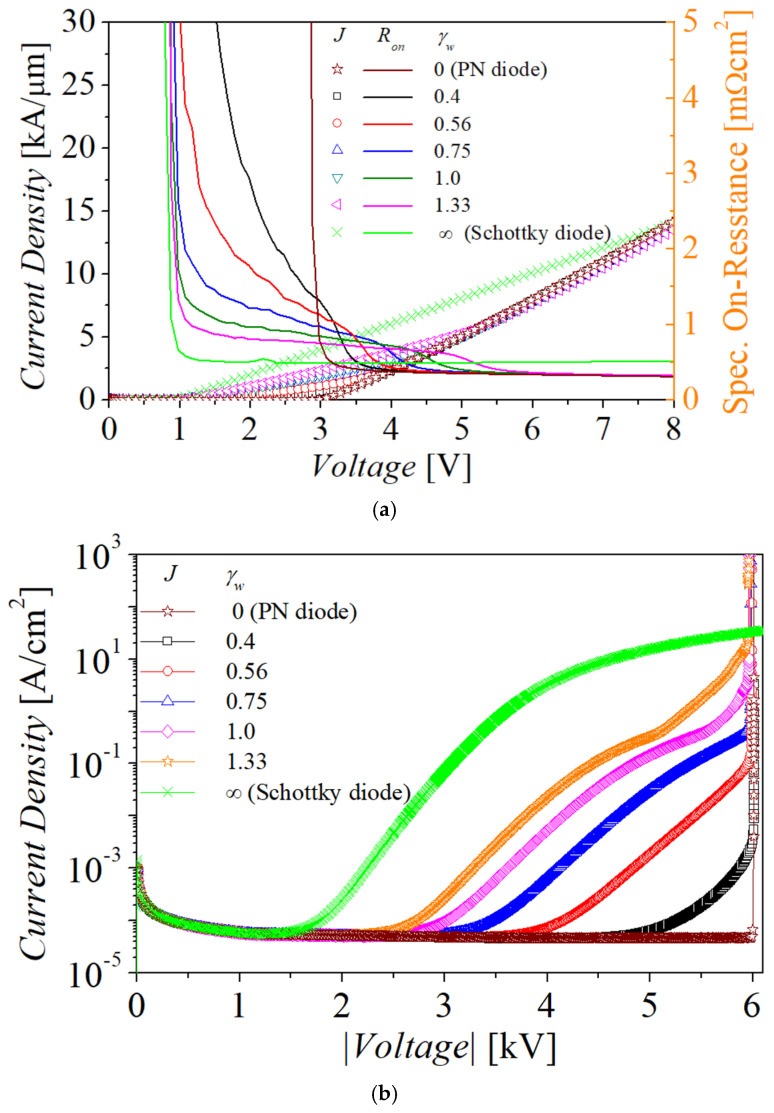
(**a**) Forward and (**b**) reverse *I-V* characteristics of the MPS diodes at a fixed *WF* of 5.2 eV and an optimized multi-drift-layer doping profile, employing seven different Schottky-to-PN contact length ratio *γ*_w_s.

Figure 6 and Table 4 summarize the forward and reverse *I-V* characteristics of the MPS diodes as a function of the Schottky-to-PN contact length ratio, defined as *γ*_w_ = *L*_SC_/*L*_PN_, together with the extracted performance metrics including *V*_knee_, *R*_on_, *BV*, *J*_leak_, and *BFOM*. To provide clear reference points, two limiting cases are also included: a conventional PN diode corresponding to *γ*_w_ = 0 and a pure Schottky diode corresponding to *γ*_w_ → ∞. For all devices, the total anode contact length is fixed as *L*_A_ = *L*_SC_ + *L*_PN_, and an identical multi-drift-layer doping profile with a fixed *WF* of 5.2 eV is employed. This configuration allows a direct and systematic investigation of the impact of lateral contact ratio on the MPS diode performance.

**Table 4:** Performance metrics extracted from Figure 6 as a function of $\gamma_{w}$.

*γ_w_* (=*L_SC_*/*L_PN_*)	*R*_on1_ [mΩ·cm^2^]	*V*_knee_ [V]	*BV* [V]	*J*_leak_ [mA/cm^2^]	*BFOM* [GW/cm^2^]
0 (PN diode)	0.31 *	3.060	6011	0.55	240.88 *
0.4 (=1.0/2.5)	1.91	2.068	6008	0.55	18.90
0.56 (=1.25/2.25)	1.40	1.588	5989	0.55	25.62
0.75 (=1.5/2.0)	1.12	1.352	5984	0.55	31.94
1.0 (=1.75/1.75)	0.92	1.232	5890	0.55	37.71
1.33 (=2.0/1.5)	0.79	1.169	5530	0.55	38.71
∞ (Schottky diode)	0.49	1.044	3813	0.60	29.67

* On-resistance at *V* = 8 V (*R*_on2_).

Figure 6a shows the forward *I-V* characteristics of the proposed MPS diodes for different values of *γ*_w_. The PN diode (*γ*_w_ = 0) exhibits a high *V*_knee_ and a steep rise in on-resistance at low forward bias, reflecting the inherent conduction loss associated with bipolar junction turn-on [11]. As *γ*_w_ increases and a Schottky conduction path is introduced, both *V*_knee_ and *R*_on_ decrease monotonically. This trend is clearly quantified in Table 4. As *γ*_w_ increases from 0.40 to 1.33, *V*_knee_ decreases from 3.06 V to 1.17 V, while *R*_on_ is reduced from 3.06 to 0.79 mΩ·cm^2^. In the limiting case of the pure Schottky diode (*γ*_w_ → ∞), the lowest *R*_on_ of 0.49 mΩ·cm^2^ is achieved, confirming the superior forward conduction capability of unipolar Schottky transport [9,14]. These results demonstrate that increasing *γ*_w_ effectively transitions the dominant conduction mechanism from bipolar PN conduction to unipolar Schottky conduction, leading to progressively reduced forward losses.

The reverse *I-V* characteristics shown in Figure 6b reveal a fundamentally different trend. The PN diode exhibits excellent reverse blocking capability with a *BV* of approximately 6.0 kV and negligible *J*_leak_. Introducing a Schottky contact through the MPS structure preserves this high *BV* across a wide range of *γ*_w_, indicating that the reverse blocking mechanism remains drift-region-limited [15,16]. For all MPS diodes with finite *γ*_w_, the *BV* remains close to 6 kV, despite substantial changes in the Schottky contact fraction. Moreover, *J*_leak_ prior to breakdown is nearly identical for all *γ*_w_ values, demonstrating that the embedded PN regions effectively shield the Schottky interface from electric-field crowding under reverse bias [13,14]. In contrast, the pure Schottky diode (*γ*_w_ → ∞) exhibits a significantly reduced *BV* of approximately 3.8 kV and a higher leakage current, reflecting Schottky-contact-limited breakdown. Importantly, the reverse current in the MPS diodes begins to increase gradually above approximately 2 kV, well before the final breakdown. This increase originates from high-field effects in the lightly doped drift region, where the expanding depletion region enhances electric fields and activates field-assisted Shockley–Read–Hall generation [28] and impact-ionization-assisted pre-avalanche carrier multiplication [29]. The insensitivity of this behavior to *γ*_w_ confirms that it is governed by the drift region rather than by Schottky-related leakage mechanisms.

The interplay between forward conduction loss and reverse blocking capability is reflected in the *BFOM*, summarized in Table 4. The PN diode achieves a *BV* but suffers from a large *R*_on_, resulting in limited *BFOM* despite excellent leakage suppression. Conversely, the pure Schottky diode offers very low *R*_on_ but a severely degraded *BV*, leading to a reduced *BFOM*. The proposed MPS diode bridges these two extremes. As *γ*_w_ increases from 0.40 to 1.33, the *BFOM* increases monotonically from 18.9 to 38.7 GW·cm^−2^, driven primarily by the strong reduction in *R*_on_ while maintaining a *BV* comparable to that of the PN diode. This result demonstrates that the MPS architecture effectively decouples forward conduction optimization from reverse blocking capability, thereby relaxing the conventional *R*_on_–*BV* trade-off.

From a design perspective, the results identify *γ*_w_ as a powerful tuning parameter for MPS diodes. While increasing $\gamma_{w}$ continuously improves forward conduction, excessively large values approaching the pure Schottky limit compromise *BV* and leakage performance. Within the investigated range, the highest *BFOM* is achieved at *γ*_w_ = 1.33, which represents an optimal balance between Schottky-dominated conduction and effective PN-assisted electric-field management.

Overall, this study demonstrates that the proposed MPS diode provides a continuous and controllable transition between PN and Schottky diode behaviors. By optimizing the *γ*_w_, the MPS structure combines the low forward loss of Schottky diodes with the high breakdown robustness of PN diodes, making it a compelling solution for high-voltage, low-loss power electronics.

**Figure 7:**
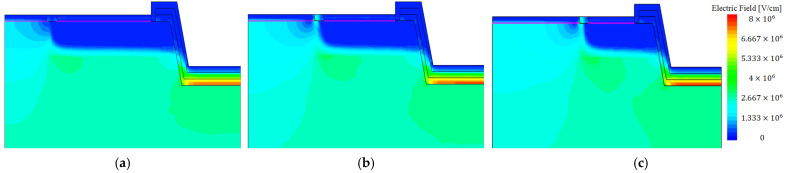
Electric-field distributions of the proposed MPS diode for three Schottky-to-PN contact length ratios: (**a**) γ_w_ = 0.4, (**b**) γ_w_ = 0.75, and (**c**) γ_w_ = 1.33, with a fixed *WF* of 5.2 eV and the optimized multi-drift-layer doping profile, under a reverse anode bias of *V* = −5 kV.

To further visualize the *γ*_w_-dependent field-shielding mechanism, Figure 7 shows the 2D electric-field distributions of the proposed MPS diode for three *γ*_w_ values (0.4, 0.75, and 1.33) under a reverse anode bias of *V* = −5 kV. As *γ*_w_ increases (i.e., the Schottky region widens while the p-GaN region narrows), the field-redistribution pattern visibly evolves: the high-field region migrates from being well-confined under the p-GaN to extending laterally toward the mesa edge. This visual evolution provides a direct physical explanation for the mild BV reduction (6008 → 5530 V) observed in Table 4 as *γ*_w_ increases.

**Figure 8:**
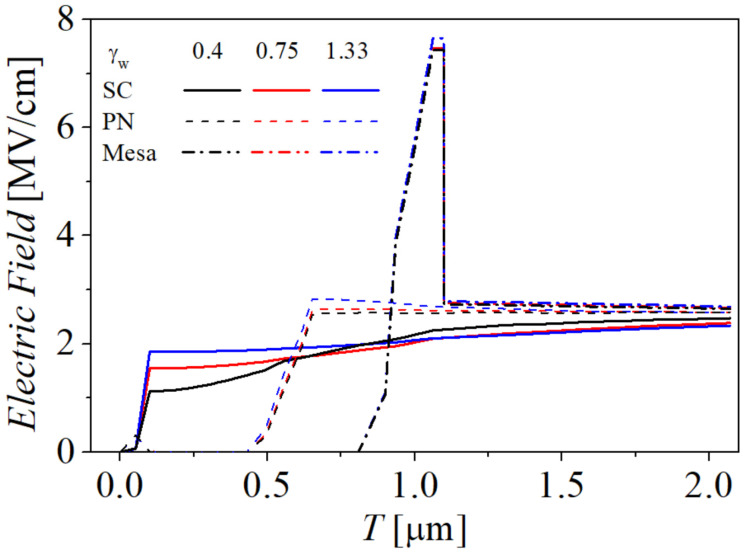
Vertical electric-field profiles of the proposed MPS diode taken along three cut-lines: through the Schottky center (*L* = 0.5 μm, denoted SC), through the p-GaN center (*L* = 2.5 μm, denoted PN), and at the mesa edge (*L* = 4.5 μm, denoted Mesa) for three values of γ_w_ (0.4, 0.75, and 1.33). A fixed *WF* of 5.2 eV and the optimized multi-drift-layer doping profile are used, under a reverse anode bias of *V* = −5 kV.

Figure 8 provides a quantitative comparison of the vertical electric-field profiles along three representative cut-lines—through the Schottky center (SC, solid lines), through the p-GaN center (PN, dashed lines), and at the mesa edge (Mesa, dash-dotted lines)—for the same three *γ*_w_ values. Three key observations emerge: (i) the field at the Schottky center remains well below 2 MV/cm for all *γ*_w_ values, quantitatively confirming the effective p-GaN shielding of the Schottky interface; (ii) the field at the p-GaN center is moderate (~2.5–3 MV/cm); and (iii) the field at the mesa edge rises sharply when *γ*_w_ increases from 0.4 to 0.75 (from ~2.5 to ~7 MV/cm), with a smaller further increase at *γ*_w_ = 1.33. This identifies the mesa edge as the dominant *BV*-limiting location at high *γ*_w_ and explains the *BV* trend in Table 4 quantitatively.

### 3.4. Temperature Dependence of the Proposed MPS Diode

**Figure 9:**
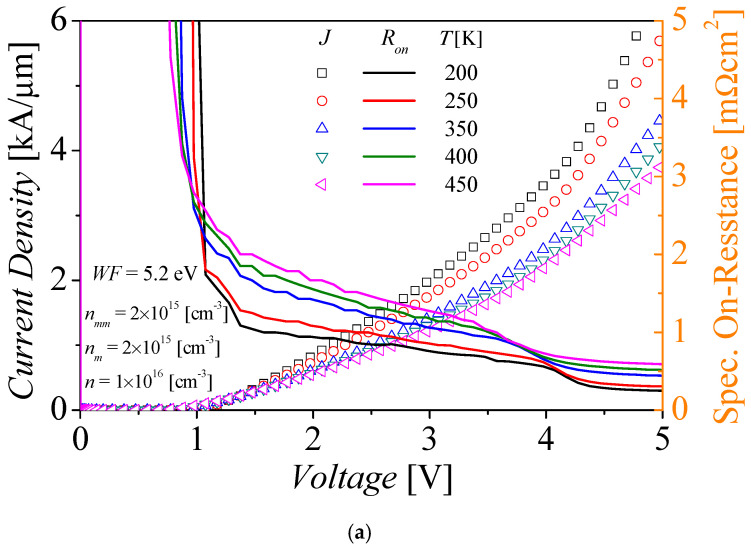

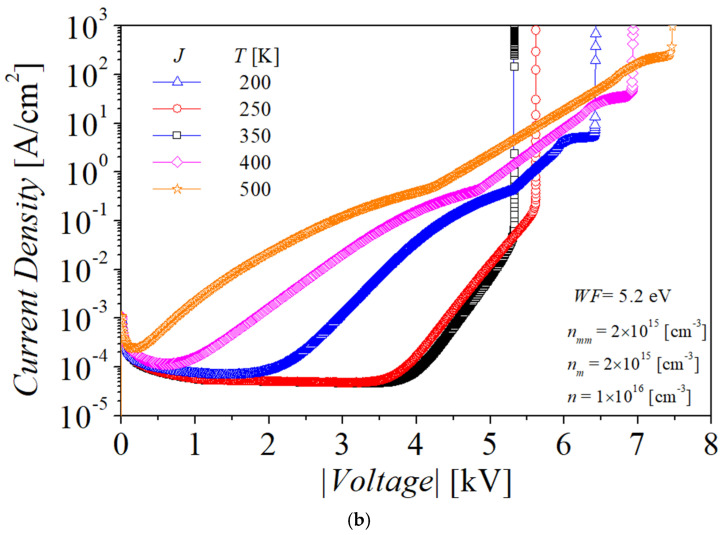
(**a**) Forward and (**b**) reverse *I-V* characteristics of the MPS diodes at a fixed *WF* of 5.2 eV and an identical multi-drift-layer doping profile, employing five different temperatures.

Figure 9 shows the temperature dependence of the forward and reverse *I-V* characteristics of the MPS diode, measured over a temperature range from 200 K to 450 K. The corresponding extracted performance metrics, including *V*_knee_, *R*_on_, *BV*, *J*_leak_, and *BFOM*, are summarized in Table 5. All devices employ an identical MPS structure with a fixed *WF* of 5.2 eV and the same optimized multi-drift-layer doping profile and Schottky contact length, ensuring that the observed variations arise solely from temperature effects.

As shown in Figure 9a, the forward *I-V* characteristics exhibit a systematic temperature dependence. With increasing temperature, the *V*_knee_ slightly decreases, reflecting the temperature-induced reduction of the effective *ϕ*_B_ and the enhanced thermionic emission at the metal–semiconductor interface [35,36]. At the same time, the extracted *R*_on_ increases monotonically with temperature, as summarized in Table 5. This increase in *R*_on_ is primarily attributed to reduced electron mobility in the GaN drift region due to enhanced phonon scattering at elevated temperatures [27,37]. Nevertheless, even at 450 K, the increase in *R*_on_ remains moderate, indicating that the unipolar Schottky conduction path in the MPS structure mitigates excessive conduction loss compared with purely bipolar devices.

Figure 9b presents the reverse *I-V* characteristics as a function of temperature. Unlike conventional Schottky diodes, which often suffer from premature breakdown and strong temperature sensitivity due to Schottky-contact-limited electric-field crowding [10], the MPS diode maintains robust reverse blocking capability across the entire temperature range. Notably, the *BV* exhibits a clear positive temperature dependence, increasing from approximately 5.32 kV at 200 K to about 7.43 kV at 450 K, as listed in Table 5. This positive temperature coefficient of *BV* indicates that breakdown in the MPS diode is dominated by avalanche multiplication in the drift region rather than by Schottky-contact-limited mechanisms [38,39]. As temperature increases, enhanced phonon scattering reduces the impact ionization coefficient, thereby requiring a higher electric field to initiate avalanche breakdown [40]. The effectiveness of the embedded PN regions in shielding the Schottky contact is further evidenced by the nearly constant reverse leakage current density prior to breakdown, which remains below 10^−3^ A/cm^2^ over the investigated temperature range. In addition, a gradual increase in reverse current is observed at high reverse voltages below breakdown, particularly above approximately 2 kV. This behavior originates from field-enhanced generation and pre-avalanche carrier multiplication within the extended depletion region of the drift layers and represents intrinsic high-field behavior rather than premature breakdown or Schottky-induced leakage [28,29].

The combined temperature dependence of *R*_on_ and *BV* is directly reflected in the evolution of *BFOM*. As shown in Table 5, despite the gradual increase in *R*_on_ with temperature, the *BFOM* improves monotonically from approximately 33.3 GW·cm^−2^ at 200 K to 37.8 GW·cm^−2^ at 450 K. This enhancement is primarily driven by the strong positive temperature coefficient of *BV*, which more than compensates for the increase in *R*_on_. This behavior demonstrates a key advantage of the MPS diode architecture. While conventional power devices often suffer from a worsened *R*_on_–*BV* trade-off at elevated temperatures, the proposed MPS diode effectively decouples forward conduction degradation from reverse blocking capability. The Schottky contact ensures low turn-on voltage and efficient carrier transport, whereas the PN-assisted drift-region-limited breakdown mechanism provides enhanced *BV* and thermal stability.

Overall, these results confirm that the proposed MPS diode exhibits robust electrical performance and improved *BFOM* over a wide temperature range, underscoring its suitability for high-voltage, high-temperature power electronic applications.

### 3.5. Switching Transient Analysis and Dynamic Performance

**Figure 10:**
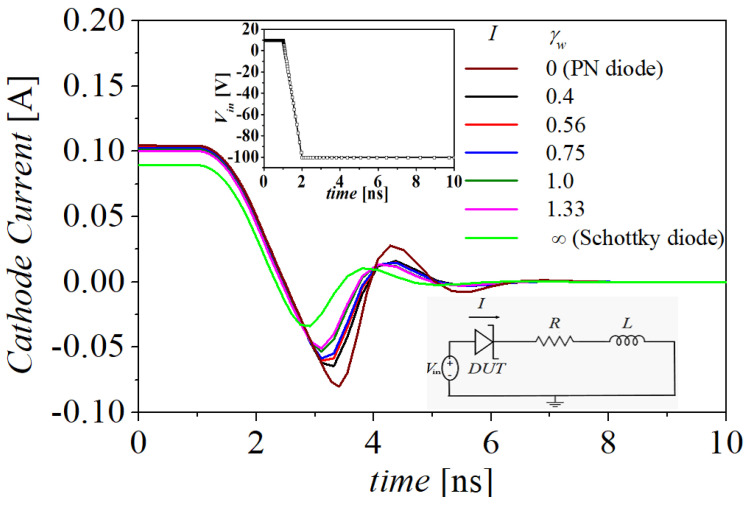
Reverse recovery switching characteristics of MPS diodes, employing seven different *γ*_w_s. The right inset shows the testing circuit with *L* = 1 μH, *R* = 1 mΩ for the reverse recovery characteristics of MPS diodes at the pulse input voltage (*V*_in_) shown in the left inset.

Figure 10 shows the reverse recovery switching characteristics of the proposed MPS diodes as a function of *γ*_w_, together with reference results for a conventional PN diode (*γ*_w_ = 0) and a pure Schottky diode (*γ*_w_ → ∞). The transient responses were obtained using mixed-mode TCAD simulations [26] that coupled a two-dimensional diode structure with an external *R*–*L* circuit, as illustrated in the right inset of Figure 10, where *L* = 1 μH and *R* = 1 mΩ. The applied pulse input voltage *V*_in_ is shown in the left inset. As shown in Figure 6, the PN diode exhibits a large reverse recovery current peak (*I*_rr(peak)_) and a pronounced recovery tail, which originate from the extraction of stored minority carriers during turn-off [41,42]. This behavior results in a relatively high *I*_rr,peak_ of 80.15 mA and a large reverse recovery charge *Q*_rr_ of 65.80 pC, as summarized in Table 6. The reverse recovery time *t*_rr_ is also relatively long, reaching 1.462 ns, which is detrimental for high-frequency switching operation. In contrast, the pure Schottky diode shows a substantially reduced reverse recovery current and a much shorter recovery interval, reflecting the absence of stored charge and the predominance of capacitive effects [43]. As a result, *I*_rr,peak_, *t*_rr_, and *Q*_rr_ are reduced to 33.84 mA, 1.140 ns, and 21.71 pC, respectively. However, this improvement in dynamic performance is accompanied by a severe degradation in *BV*, which decreases to 3.81 kV, highlighting the intrinsic trade-off between switching speed and blocking capability in conventional Schottky diodes.

The proposed MPS diodes exhibit an intermediate and controllable reverse recovery behavior between these two extremes. As *γ*_w_ increases, a systematic reduction in reverse recovery parameters is observed. Specifically, when *γ*_w_ increases from 0.4 to 1.33, *I*_rr,peak_ decreases from 64.44 mA to 50.90 mA, while the reverse recovery time *t*_rr_ is reduced from 1.467 ns to 1.321 ns. Correspondingly, *Q*_rr_ decreases from 52.08 pC to 36.86 pC, representing a reduction of approximately 29% compared with the PN diode case. This improvement is attributed to the enhanced contribution of the Schottky conduction path, which suppresses minority-carrier injection, while the embedded PN regions continue to provide effective electric-field shielding under reverse bias [14,15].

Notably, the MPS diode with *γ*_w_ = 1.0 and 1.33 achieves a favorable balance between dynamic and blocking performance. For *γ*_w_ = 1.33, the device maintains a high *BV* of 5.53 kV while exhibiting a significantly reduced *Q*_rr_ of 36.86 pC and a shortened *t*_rr_ of 1.321 ns. Compared with the PN diode, this corresponds to a marked reduction in reverse recovery loss without sacrificing high-voltage robustness. These characteristics are particularly advantageous for high-frequency power systems, where *Q*_rr_ and *t*_rr_ directly impact switching loss, current overshoot, and electromagnetic interference [43,44].

Overall, the results in Figure 10 and Table 6 clearly demonstrate that the Schottky-to-PN contact length ratio is a critical design parameter governing the reverse recovery dynamics of the MPS diode. By appropriately selecting *γ*_w_, the proposed MPS structure effectively alleviates the conventional trade-off between fast switching and high breakdown voltage, enabling improved dynamic performance while preserving PN-like blocking capability. This balance underscores the suitability of the proposed MPS diodes for high-voltage, high-frequency power electronic applications.

### 3.6. Drift-Layer Thickness Optimization and Joint Doping-Thickness Optimum

While Section 3.2 established the optimal doping concentrations under fixed default drift-layer thicknesses, the layer thicknesses themselves represent an additional degree of design freedom that we had not yet exploited. To complete the design optimization, this new section systematically varies the three drift-layer thicknesses (*T*_nmm_, *T*_nm_, *T*_n_) for the two leading doping profiles from Section 3.2—the uniform profile (*n*_mm_ = *n*_m_ = 2 × 10^15^, *n* = 1 × 10^16^ cm^−3^) and the graded profile (*n*_mm_ = 2 × 10^15^, *n*_m_ = 5 × 10^15^, *n* = 1 × 10^16^ cm^−3^)—at the fixed optimum *WF* = 5.2 eV. The simulation set is organized into two complementary groups: Group A (*T*_nmm_ fixed at 4.49 μm; *T*_nm_ and *T*_n_ traded off at constant *T*_nm_ + *T*_n_ = 25 μm) and Group B (*T*_n_ fixed at 5 μm; *T*_nmm_ and *T*_nm_ traded off at constant *T*_nmm_ + *T*_nm_ = 24.49 μm).

For the uniform doping profile, as shown in Table 7, three key findings emerge. (1) The optimum is at (*T*_nmm_, *T*_nm_, *T*_n_) = (4.49, 5, 20) μm, achieving *BFOM* = 51.05 GW/cm^2^ (*BV* = 6626 V, *R*_on1_ = 0.86 mΩ·cm^2^)—a substantial improvement over the default configuration (*T*__nm_ = 20, *T*_n_ = 5: *BFOM* = 31.97 GW/cm^2^). Remarkably, this new configuration simultaneously improves both metrics: *BV* increases by +11% and *R*_on1_ decreases by −23%. (2) *T*_n_ is the dominant thickness parameter. The monotonic trend in Group A (*BFOM* = 31.97 → 42.37 → 46.37 → 51.05 GW/cm^2^ as *T*_n_ increases from 5 to 20 μm) clearly identifies *T*_n_ as the dominant knob. (3) *T*_nmm_ and *T*_nm_ are electrically interchangeable when *n*_mm_ = *n*_m_. The Group B rows in Table 7 (4.49/20/5, 8.49/16/5, 14.49/10/5) yield identical *V*_knee_, *R*_on1_, *BV*, and *BFOM*, because *n*_mm_ = *n*_m_ makes the n^− −^ and n^−^ layers electrically indistinguishable; only their combined thickness (*T*_nmm_ + *T*_nm_) matters.

For the graded doping profile, as shown in Table 8, the same thickness pattern (large *T*_n_ and small *T*_nm_) achieves an even better result: *BFOM* = 55.36 GW/cm^2^ (*BV* = 6613 V, *R*_on1_ = 0.79 mΩ·cm^2^) at (*T*_nmm_, *T*_nm_, *T*_n_) = (4.49, 5, 20) μm. This is the overall optimum of this work—+73% improvement over the doping-only optimum reported in Section 3.2 (31.97 GW/cm^2^). The improvement comes mainly from the lower *R*_on_ (0.79 vs. 0.86 mΩ·cm^2^), enabled by the more conductive *n*^−^ layer (5 × 10^15^ vs. 2 × 10^15^ cm^−3^).

Moreover, Table 8 reveals two important physical distinctions from the uniform-doping case. First, *T*_nmm_ and *T*_nm_ are no longer interchangeable—the Group B rows of Table 8 (4.49/20/5, 8.49/16/5, 14.49/10/5) yield strongly different *BFOM* (24.18 → 26.51 → 44.47 GW/cm^2^), because *n*_mm_ ≠ *n*_m_ makes the two layers electrically distinguishable. Second, increasing *T*_nmm_ (the lightly-doped layer adjacent to the p-GaN junction) substantially raises *BV* (4560 → 6635 V in Group B), because the depletion peak-field zone has more lightly-doped material available before reaching the higher-doped *n*^−^ layer.

Physical interpretation—punch-through/field-stop principle. The performance gain at large *T*_n_ can be understood as follows. The lightly-doped (*n*^− −^ + *n*^−^) region acts as the primary blocking region, while the more heavily doped n region (5× higher doping) acts as a quasi-field-stop layer. In the default configuration (*T*_n_ = 5 μm), the light region (24.49 μm thick) supports most of the reverse voltage with a strongly triangular electric-field profile, and the thin n layer plays only a minor role. In the new optimum (*T*_n_ = 20 μm, light region = 9.49 μm), the depletion punches through the thin light region at lower reverse voltage and then extends into the n layer. The wider n layer thus contributes a large additional voltage-supporting region (raising *BV*), while simultaneously providing a lower-resistivity bulk current path (reducing *R*_on_). This is precisely the punch-through/field-stop principle that has been successfully exploited in IGBTs and SiC power diodes for decades [45,46], and we have now established it as the governing design principle for the proposed GaN MPS diode.

A major paper-level finding—doping-thickness coupling. In the original Section 3.2, the doping was optimized at the fixed default thicknesses; under that constraint, the uniform profile (2, 2, 10) × 10^15^ was found to be best (*BFOM* = 31.97), and the graded profile (2, 5, 10) × 10^15^ appeared suboptimal (*BFOM* = 24.18). However, the present joint study shows that those ranking reverses when the thickness is also optimized: the graded profile becomes the overall best (*BFOM* = 55.36), exceeding the uniform-profile thickness-optimum (*BFOM* = 51.05). The original Section 3.2 conclusion is therefore conditional on the default thickness, and the true optimum requires joint doping-thickness optimization. To our knowledge, this coupled doping-thickness design space has not been previously reported for GaN MPS diodes.

In summary, the overall optimum of the proposed MPS diode is: doping (*n*_mm_, *n*_m_, *n*) = (2 × 10^15^, 5 × 10^15^, 1 × 10^16^) cm^−3^; thickness (*T*_nmm_, *T*_nm_, *T*_n_) = (4.49, 5, 20) μm; performance *BV* = 6.61 kV, *R*_on1_ = 0.79 mΩ·cm^2^, *BFOM* = 55.36 GW·cm^−2^. This optimum is also fully consistent with the lower-bound finding of Section 3.2 that *n*_mm_ = 2 × 10^15^ cm^−3^ represents a practical lower limit for the lightly-doped layer.

## 4. Conclusions

In this work, a GaN vertical MPS diode incorporating a three-sub-layer n-type drift region, composite SiO_2_/Si_3_N_4_ passivation, and anode field-plate termination has been designed and comprehensively analyzed through 2D TCAD simulations. The following conclusions can be drawn:

(1) Work function dependence: The Schottky metal work function governs the trade-off between *V*_knee_ and *BV*. While a lower work function (*WF* = 4.5 eV) achieves the smallest *V*_knee_ of 0.693 V, the breakdown voltage is severely limited (152 V) due to a low *ϕ*_B_. A higher work function (*WF* = 5.2 eV) simultaneously elevates *BV* to over 3.3 kV and suppresses reverse leakage current, making it the preferred choice for high-voltage MPS diodes.
(2) Multi-drift-layer optimization: At fixed default thicknesses, the optimum doping (*n*_mm_ = 2 × 10^15^, *n*_m_ = 2 × 10^15^, *n* = 1 × 10^16^ cm^−3^) achieves *BFOM* = 31.97 GW·cm^−2^. Extending the low-doping sweep down to 1 × 10^14^ cm^−3^ confirms that 2 × 10^15^ cm^−3^ is a practical lower bound: BV saturates while *R*_on_ increases ~19× and BFOM collapses 16× as doping decreases. The joint doping–thickness optimization in Section 3.6 identifies a new overall optimum: graded doping (*n*_mm_ = 2 × 10^15^, *n*_m_ = 5 × 10^15^, n = 1 × 10^16^ cm^−3^) combined with thicknesses (*T*_nmm_, *T*_nm_, *T*_n_) = (4.49, 5, 20) μm, achieving *BFOM* = 55.36 GW·cm^−2^ (*BV* = 6.61 kV, *R*_on_ = 0.79 mΩ·cm^2^)—a +73% improvement. This is governed by the punch-through/field-stop design principle and demonstrates that doping and thickness must be co-optimized rather than optimized sequentially.
(3) Schottky contact length ratio: Increasing γ_w_ from 0.40 to 1.33 monotonically reduces *V*_knee_ and *R*_on_ while maintaining *BV* ≈ 6 kV, resulting in a *BFOM* improvement from 18.90 to 38.71 GW·cm^−2^. The value *γ*_w_ = 1.33 is identified as the optimal design point, providing the highest *BFOM* without sacrificing the PN-assisted reverse blocking capability.
(4) Temperature stability: The MPS diode exhibits robust performance over a wide temperature range (200–450 K). The *BV* increases with temperature due to the positive temperature coefficient of avalanche breakdown, and the *BFOM* improves monotonically from 33.31 to 37.82 GW·cm^−2^, confirming thermal stability superior to conventional Schottky diodes.
(5) Switching performance: Mixed-mode TCAD simulations demonstrate that increasing *γ*_w_ reduces *Q*_rr_ by approximately 29% relative to the PN diode reference, while maintaining PN-like *BV*. The proposed MPS structure at *γ*_w_ = 1.33 achieves an excellent balance between fast switching (*Q*_rr_ = 36.86 pC, *t*_rr_ = 1.321 ns) and high blocking capability (*BV* = 5.53 kV).
(6) Internal distribution analysis: 2D contour plots of the electrostatic potential, current density, and reverse-bias electric field (Figure 3, Figure 4, Figure 7 and Figure 8) directly visualize the dual-path forward conduction and the p-GaN field-shielding mechanism. The cut-line profiles in Figure 8 identify the mesa edge as the dominant *BV*-limiting location at high *γ*_w_.

Overall, the proposed GaN vertical MPS diode with multi-drift-layer and field-plate termination effectively decouples forward conduction loss from reverse blocking capability, substantially relaxing the conventional *R*_on_–*BV* trade-off. The identified design guidelines provide a practical roadmap for realizing high-voltage, high-efficiency GaN power rectifiers for next-generation power electronic systems. These improvements are expected to enhance the high-frequency figure of merit, highlighting the advantage of the proposed MPS diode in switching applications.

## Figures and Tables

**Table 1:** Structural parameters of the proposed GaN MPS diode.

Parameter	Value (Default)	Unit	Description
*T*_p+_	0.01	μm	p^+^-GaN layer thickness
*T*_p_	0.5	μm	p-GaN layer thickness
*T*_nmm_	Variable (4.49)	μm	n^− −^-GaN layer thickness
*T*_nm_	Variable (20)	μm	n^−^-GaN layer thickness
*T*_n_	Variable (5)	μm	n-GaN layer thickness
*T*_n+_	2	μm	n^+^-GaN substrate thickness
*T*_Si3N4_	0.1	μm	Si_3_N_4_ passivation thickness
*T*_SiO2_	0.1	μm	SiO_2_ passivation thickness
*L*_SC_	Variable (1.5)	μm	Schottky contact length
*L*_PN_	Variable (2.0)	μm	p-GaN region length
*L*_S_	0.25	μm	Spacing length
*L*_SD_	1.25	μm	Field-plate extension

**Table 5:** Performance metrics extracted from Figure 9 as a function of temperature.

*T* [K]	*R*_on1_ [mΩ·cm^2^]	*R*_on2_ [mΩ·cm^2^]	*V*_knee_ [V]	*BV* [V]	*J*_leak_ [mA/cm^2^]	*BFOM* [GW/cm^2^]
200	0.85	0.187	1.351	5321	0.54	33.31
250	0.99	0.247	1.351	5616	0.55	31.86
300	1.12	0.317	1.351	5984	0.55	31.97
350	1.23	0.388	1.351	6441	0.56	33.72
400	1.35	0.461	1.344	6945	0.59	35.73
450	1.48	0.534	1.328	7481	0.69	37.82

**Table 6:** Performance metrics extracted from Figure 10 as a function of *γ*_w_.

*γ_w_* (=*L_SC_*/*L_PN_*)	*R*_on1_ [mΩ·cm^2^]	*BV* [kV]	|*I*_rr(peak)_| [mA]	*t*_rr_ [ns]	*Q*_rr_ [pC]
0 (PN diode)	0.31 *	6011	80.15	1.462	65.80
0.4 (=1.0/2.5)	1.91	6008	64.44	1.467	52.08
0.56 (=1.25/2.25)	1.40	5989	60.24	1.424	49.22
0.75 (=1.5/2.0)	1.12	5984	58.56	1.405	47.09
1.0 (=1.75/1.75)	0.92	5890	53.48	1.331	38.40
1.33 (=2.0/1.5)	0.79	5530	50.90	1.321	36.86
∞ (Schottky diode)	0.49	3813	33.84	1.140	21.71

* On-resistance at *V* = 8 V (*R*_on2_).

**Table 7:** BFOM performance comparison across thickness of multi-drift-layer doping configurations in the MPS diode (*WF* = 5.2 eV, *n*_mm_ = 2 × 10^15^, *n*_m_ = 2 × 10^15^, and *n* = 1 × 10^16^ cm^−3^).

*T*_nmm_ [µm]	*T*_nm_ [µm]	*T*_nm_ [µm]	*V*_knee_ [V]	*R*_on1_ [mΩ·cm^2^]	*BV* [V]	*J*_leak_ [mA/cm^2^]	*BFOM* [GW/cm^2^]
2.49	22	7	1.352	1.12	5979	0.55	31.92
4.49	5	20	1.319	0.86	6626	0.55	51.05
4.49	10	15	1.328	0.95	6637	0.55	46.37
4.49	15	10	1.341	1.03	6606	0.55	42.37
4.49	20	5	1.352	1.12	5984	0.55	31.97
8.49	16	5	1.352	1.12	5984	0.55	31.97
14.49	10	5	1.352	1.12	5984	0.55	31.97

**Table 8:** *BFOM* performance comparison across thickness of multi-drift-layer doping configurations in the MPS diode (*WF* = 5.2 eV, *n*_mm_ = 2 × 10^15^, *n*_m_ = 5 × 10^15^, and *n* = 1 × 10^16^ cm^−3^).

*T*_nmm_ [µm]	*T*_nm_ [µm]	*T*_nm_ [µm]	*V*_knee_ [V]	*R*_on1_ [mΩ·cm^2^]	*BV* [V]	*J*_leak_ [mA/cm^2^]	*BFOM* [GW/cm^2^]
2.49	22	7	1.314	0.83	6591	0.55	52.34
4.49	5	20	1.310	0.79	6613	0.55	55.36
4.49	10	15	1.313	0.81	6634	0.55	54.33
4.49	15	10	1.316	0.84	6622	0.55	52.20
4.49	20	5	1.319	0.86	4560	0.55	24.18
8.49	16	5	1.324	0.91	4912	0.55	26.51
14.49	10	5	1.335	0.99	6635	0.55	44.47

## Data Availability

The original contributions presented in this study are included in the article. Further inquiries can be directed to the corresponding author.
